# Sex differences in vulnerability to tau pathology: Impact on cognitive decline

**DOI:** 10.1002/alz.70634

**Published:** 2025-10-08

**Authors:** Yashar Zeighami, Cecilia Tremblay, Mahsa Dadar

**Affiliations:** ^1^ Department of Psychiatry McGill University Montréal Québec Canada; ^2^ Douglas Mental Health University Institute Montréal Québec Canada

**Keywords:** Alzheimer's disease pathology, amyloid plaques, biomarkers, cerebrospinal fluid, cognitive decline, neurodegeneration, positron emission tomography, sex, tau tangles

## Abstract

**INTRODUCTION:**

Although the link between the presence of amyloid and tau pathologies, neurodegeneration, and cognitive decline in aging individuals is established, it is less clear whether there are sex differences in vulnerability to these pathologies.

**METHODS:**

A total of 1464 participants (7168 longitudinal assessments, 4.77 ± 3.78 years of follow‐up) were included from the National Alzheimer's Coordinating Center (NACC) database. Longitudinal mixed effects and mediation models examined the sex differences across cognitive decline trajectories of amyloid (A), tau (T), and neurodegeneration (N) groups.

**RESULTS:**

A^+^T^−^ males showed faster cognitive decline compared to A^+^T^−^ females (*p* < 0.005), whereas A^+^T^+^ females showed steeper cognitive decline compared to A^+^T^+^ males (*p* < 0.0001). In addition, sex marginally moderated the mediating effect of tau on the relationship between amyloid and cognitive decline (*p* = 0.046).

**DISCUSSION:**

Sex differences in vulnerability to tau pathology in the presence of amyloid can shape cognitive decline trajectories.

**Highlights:**

- A^+^T^−^ males showed faster cognitive decline compared to A^+^T^−^ females.
- A^+^T^+^ females showed faster cognitive decline compared to A^+^T^+^ males.
- Tau status significantly mediated the relationship between amyloid status and cognitive decline.
- Sex marginally moderated the mediating relationship between amyloid, tau, and cognitive decline.
- The findings point to sex differences in the impact of tau pathology on cognition.

## 1 BACKGROUND

Alzheimer's disease (AD) is the most common neurodegenerative disease and cause of dementia in the aging population. Based on the definition of the National Institute on Aging–Alzheimer's Association (NIA‐AA) Alzheimer's diagnostic framework to biologically define AD, it is characterized by the presence of three distinct biomarkers: amyloid beta (Aβ) deposition (A), pathological tau (T), and neurodegeneration (N).^1^, ^2^ Clinically, positivity on amyloid and tau markers can be established through amyloid and tau positron emission tomography (PET) or cerebrospinal fluid (CSF) assessments, whereas neurodegeneration can be determined based on magnetic resonance imaging (MRI) or fluorodeoxyglucose (FDG) PET findings^2^. Longitudinal trajectories of AD amyloid‐tau‐neurodegeneration (ATN) biomarkers have been assessed in older adults, with the most common pattern being the amyloid pathology biomarker that emerges first.^2^ Specifically, individuals who are negative on all three markers (A^−^ T^−^ N^−^) are considered as normal or without AD pathology, individuals that are positive on amyloid and negative on tau and neurodegeneration (A^+^ T^−^ N^−^) are considered to be at earlier stages of the disease, and individuals that are positive on amyloid and tau (A^+^ T^+^ N^−^ or A^+^ T^+^ N^+^) are considered as having AD (earlier or later stages, respectively). Finally, individuals that are negative on amyloid but positive on tau or neurodegeneration (A^−^ T^+^ N^−^, A^−^ T^−^ N^+^, or A^−^ T^+^ N^+^) are considered to have non‐AD pathologies, and individuals that are positive on amyloid and neurodegeneration but negative on tau are considered as having AD pathology as well as other non‐AD pathology (A^+^T^−^N^+^) that contributes to neurodegeneration.^2^, ^3^

A growing body of research on sex differences in AD points to significantly higher prevalence and burden of tau pathology and cognitive deficits in females compared to males.^4^, ^5^, ^6^, ^7^ Sex‐specific differences in pathology burden might also have implications for disease management and treatment, as sex differences in treatment efficiency have been reported recently.^8^, ^9^ Finally, although previous studies suggest a mediating effect of tau pathology on the associations between amyloid burden and cognitive decline,^10^, ^11^, ^12^, ^13^ it is unclear whether these associations differ between sexes. As such, given the accumulating literature pointing to sex differences in AD pathology burden and cognitive decline trajectories,^3^, ^14^, ^15^, ^16^, ^17^, ^18^ more studies are needed to disentangle the effects of sex on the associations between amyloid and tau biomarkers and cognitive decline.

This study aimed to assess the sex differences in longitudinal cognitive trajectories, specifically disentangling the contribution of amyloid, tau, and neurodegeneration, by comparing ATN biomarker–based groups using a large cohort of aged individuals along the AD continuum, from cognitively unimpaired to AD dementia. To do so, we took advantage of the large number of participants with ATN biomarker status information as well as long‐term longitudinal cognitive assessments available from the National Alzheimer's Coordinating Center (NACC)^19^, ^20^ database. We hypothesized that amyloid‐ and tau‐positive biomarker groups with neurodegeneration would show a steeper cognitive decline followed by groups positive for amyloid without neurodegeneration or tau pathology, and we expected to find a steeper cognitive decline in females, especially in groups with tau‐positive biomarkers.

**RESEARCH IN CONTEXT**

1. **Systematic review**: The authors reviewed the literature on the associations between the presence of amyloid and tau pathologies and neurodegeneration and cognitive decline in individuals on the Alzheimer's disease (AD) continuum, using traditional sources such as PubMed. Although previous research has highlighted the impact of the presence of these pathologies on exacerbating cognitive decline in these populations as well as a mediation relationship between amyloid, tau, and cognitive decline, it is unclear whether these relationships are sex differentiated.
2. **Interpretation**: Our main findings revealed a specific dichotomic sex difference between tau positive versus tau negative biomarker groups that had positive amyloid biomarker status. Specifically, in the A^+^T^−^ group, males showed a faster cognitive decline than females in contrast to the A^+^T^+^ group, in which females showed a steeper cognitive decline. Furthermore, sex moderated the mediation relationship between amyloid, tau, and cognitive decline. These results suggest differences in the vulnerability to tau pathology in participants with positive amyloid biomarkers and may have important clinical implications for the development of sex‐specific treatments in AD.
3. **Future directions**: Future research should extend these findings by incorporating other contributing factors such as vascular pathology and inflammatory markers into the ATN framework.

## 2 METHODS

Data were obtained from the NACC (https://naccdata.org/; September 2024 data freeze) database.^19^, ^20^, ^21^, ^22^ Ethics approval was obtained from the participants by the local institutional review board from each NIA Alzheimer's Disease Research Center (ADRC) that contributed the data to NACC. Yes/no indicators of elevated amyloid and tau based on PET or CSF were provided by NACC ADRCs based on local interpretations of PET or CSF results (https://files.alz.washington.edu/documentation/uds3‐tip‐ded.pdf). Briefly, PET positivity was established based on visual interpretation of the images following image preparation (reorientation and color scaling), in compliance with U.S. Food and Drug Administration (FDA)– and European Medicines Agency (EMA)–approved guidelines for clinical use.^23^ The CSF biomarkers for amyloid and tau were analyzed using enzyme‐linked immunosorbent assay (ELISA) or Luminex multiplex xMAP assay protocols, and positivity was established based on assay specific thresholds.^24^ Participants were included if they had demographics information as well as PET‐ or CSF‐based amyloid (*N* = 1074 for PET and *N *= 390 for CSF) and tau (*N* = 886 for PET and *N* = 578 for CSF) status and visual assessment of hippocampal atrophy (*N* = 1464) available. These binary values reflecting abnormality on amyloid and tau and hippocampal atrophy were used to derive ATN classification for all participants. Clinical Dementia Rating Sum of Boxes (CDR‐SB) scale scores were used as a measure of global cognition. Exclusion criteria included missing ATN information, clinical diagnosis of non‐AD neurological disorders (e.g., Lewy body dementia and frontotemporal dementia), participants with ages below 55 or above 95, and participants without longitudinal follow‐up assessments including CDR‐SB information available. Furthermore, due to the small number of participants with A^−^ T^+^ N^+^ (2 females and 12 males), this group was not included in the analyses. This resulted in a total of 1464 participants with 7168 longitudinal timepoints included (average follow‐up: 4.77 ± 3.78 years, 4.9 ± 3.2 visits). Table 1 summarizes the demographics information of the included participants. Names of the specific variables used are summarized in Table S1.

**TABLE 1:** Baseline characteristics of the participants included in this study.

	Females *N* = 772	Males *N* = 692	*p*‐values
Age	67.66 ± 7.61	69.40 ± 7.86	< 0.001
*APOE* ε4 allele, *N* (%)	638 available	570 available	
0	319 (50%)	288 (50%)	0.39
1	249 (39%)	233 (41%)	
2	70 (11%)	49 (9%)	
Education	15.96 ± 2.7	16.80 ± 2.6	< 0.001
Amyloid positive (A^+^), *N* (%)	352 (50%)	390 (56%)	< 0.001
Tau positive (T^+^), *N* (%)	297 (42%)	325 (47%)	0.001
Neurodegeneration positive (N^+^), *N* (%)	208 (30%)	295 (42%)	<0.001
CDR‐SB	1.53 ± 2.47	1.90 ± 2.49	0.004
MoCA	22.59 ± 6.06	22.14 ± 5.37	0.22
Trail Making Test Part A	39.05 ± 28.51	42.12 ± 28.37	0.046
Trail Making Test Part B	99.63 ± 70.7	113.92 ± 74.93	< 0.001
Animals	18.76 ± 6.78	17.73 ± 6.76	< 0.005
Vegetables	13.75 ± 5.40	10.37 ± 4.77	< 0.0001
Diagnosis, *N* (%)			
NC	393 (51%)	213 (31%)	
MCI	204 (26%)	305 (44%)	<0.0001
AD	175 (23%)	174 (25%)	
Thal amyloid phase (total sample)	(42)	(70)	0.235
0	4	2	
1	2	1	
2	0	3	
3	3	5	
4	6	18	
5	27	41	
CERAD amyloid score (total sample)	(42)	(70)	0.334
0	7	7	
1	0	4	
2	3	6	
3	32	53	
Braak tau stage (total sample)	(41)	(68)	0.051
0	2	0	
1	2	1	
2	0	7	
3	3	1	
4	3	2	
5	8	12	
6	23	45	

Abbreviations: AD, Alzheimer's disease; *APOE*, apolipoprotein E; CDR‐SB, Clinical Dementia Rating–Sum of Boxes; CERAD, Consortium to Establish a Registry for Alzheimer's Disease; MCI, mild cognitive impairment; NC, normal cognition; MoCA, Montreal Cognitive Assessment.

### 2.1 Statistical analyses

Baseline demographic characteristics were compared across sexes using independent sample *t*‐tests for continuous measures and chi‐square (𝜒^2^) tests for categorical measures. The following longitudinal mixed‐effects models were used to examine the differences in rates of cognitive decline across ATN groups (Model 1) as well as potential sex differences in the rates of decline within each ATN group (Model 2):

(1) $$\begin{array}{ccc} & & \mathrm{Cognition}∼\mathrm{Time}\;\mathrm{from}\;\mathrm{Baseline}:\mathrm{ATN}+\mathrm{Time}\;\mathrm{from}\;\mathrm{Baseline}\\ & & \;+\;\mathrm{ATN}+{\mathrm{Age}}_{\mathrm{bl}}+\mathrm{Education}+\left(1|\mathrm{ID}\right) \end{array}$$

(2) $$\begin{array}{ccc} & & \mathrm{Cognition}∼\mathrm{Time}\;\mathrm{from}\;\mathrm{Baseline}:\mathrm{ATN}:\mathrm{Sex}+\mathrm{Time}\;\mathrm{from}\;\mathrm{Baseline}:\\ & & \;\mathrm{ATN}+\mathrm{Time}\;\mathrm{from}\;\mathrm{Baseline}+\mathrm{ATN}+\mathrm{Time}\;\mathrm{from}\;\mathrm{Baseline}:\mathrm{Sex}\\ & & \;+\;\mathrm{ATN}:\mathrm{Sex}+\mathrm{Sex}+\mathrm{Ag}e_{\mathrm{bl}}+\mathrm{Education}+\left(1|\mathrm{ID}\right) \end{array}$$

where cognition indicates CDR‐SB scores, time from baseline indicates the time between each longitudinal time point and the baseline clinical assessment visit, and ATN indicates a categorical variable reflecting positivity on amyloid, tau, and neurodegeneration. Sex is a categorical variable contrasting males versus females. Age at baseline (Age_bl_) and education were included as continuous fixed covariates in all models. For Model 1, the variable of interest was **Time from Baseline: ATN**, reflecting the potential differences in rates of cognitive decline across groups of interest.

For Model 2, the variable of interest was **Time from Baseline: ATN: Sex**, reflecting potential sex differences in rates of cognitive decline across the groups of interest. To ensure that potential differences in age (as the female participants were younger than the male participants in the full sample) and clinical diagnoses of the participants included (as there was a higher proportion of cognitively normal females compared to males in the full sample) did not impact the results, the analyses were repeated in an age‐ and diagnosis‐matched subset of the dataset (*N* = 1146, 573 females, 573 males).

Similar analyses (i.e., Models 1 and 2) were performed for other cognitive assessment scores that had sufficient data available, evaluating the following cognitive domains: (1) global cognition: Montreal Cognitive Assessment (MoCA, *N* = 1182, 4042 longitudinal assessments); (2) executive functioning: Trail Making Test (TMT) Parts A (*N* = 1242, 5420 longitudinal assessments) and B (*N* = 1127, 5045 longitudinal assessments); and (3) semantic functioning: Animals (*N* = 1357, 6294 longitudinal assessments) and Vegetables (*N* = 1346, 6249 longitudinal assessments). The estimates for MoCA, Animals, and Vegetables were multiplied by (–1) so that positive estimates indicate worse cognitive performance for all tests.

To assess whether there were potential differences in severity of pathologies between males and females (e.g., whether T^+^ females had a greater burden of tau tangles than males), we further examined the sex differences in post‐mortem amyloid and tau levels, using Thal amyloid phase, Consortium to Establish a Registry for Alzheimer's Disease (CERAD) amyloid score, and Braak tau stage (see original variable names in Table S1) in the subset of the participants that had post‐mortem pathology assessments available (N_Braak_: 68 males and 41 females; N_Thal_: 70 males and 42 females; N_CERAD_: 70 males and 42 females). The following ordinal logistic regression models were used for these analyses, adjusting for age at time of death.

(3) Braakstage∼Sex:Taustatus+Sex+Taustatus+ageatdeath

(4) $$\begin{array}{ccc} & & \mathrm{Thal}\;\mathrm{phase}/\mathrm{CERAD}∼\;\mathrm{Sex}:\mathrm{Amyloid}\;\mathrm{status}+\mathrm{Sex}+\mathrm{Amyloid}\;\mathrm{status}\\ & & \;+\;\mathrm{age}\;\mathrm{at}\;\mathrm{death} \end{array}$$

where amyloid and tau status denote in vivo A and T status, and Braak, Thal, and CERAD denote the standard post‐mortem pathology staging scores of the same participants. The variables of interest included sex and sex interaction with amyloid or tau status.

Finally, we examined whether there are sex differences in the mediating effect of tau positivity on the associations between amyloid positivity and cognitive decline. We first confirmed the mediating relationships between amyloid, tau, and cognitive decline by performing a mediation analysis, with amyloid status as the independent variable, rate of cognitive decline as the dependent variable, and tau status as the mediating variable. Age and education were included as covariates. Rate of cognitive decline was estimated based on the random slopes of the following mixed effects models, similar to previous studies^25^, ^26^ in the literature:

(5) $$\mathrm{Cognition}∼1+\mathrm{Time}\;\mathrm{from}\;\mathrm{Baseline}+\left(\mathrm{Time}\;\mathrm{from}\;\mathrm{Baseline}\;|\;\mathrm{ID}\right)$$

Sex was then examined as a potential moderator for this mediation effect. For both analyses, we used 1000 bootstrapped resamples to estimate the confidence intervals (CIs). All statistical analyses were performed in R software version 4.3.3 (2024‐02‐29). The matched subsample was produced using MatchIt version 4.7.1^27^, ^28^ R package. lme4 version 1.1‐36^29^, ^30^ and lmerTest version 3.1‐3 ^31^ R packages were used for mixed‐effects modeling analyses. “lavaan” version 0.6‐19^32^ R package was used for the mediation and moderation analyses.

## 3 RESULTS

Female participants were younger and had lower levels of education (*p* < 0.001). Furthermore, fewer females were A^+^, T^+^, or N^+^. Females also had lower CDR‐SB scale scores at baseline (*p* = 0.004), and a greater proportion of them were classified as cognitively normal at the baseline visit assessment, whereas a greater proportion of males were classified in the mild cognitive impairment (MCI) group (*p* < 0.0001). As expected, there were no statistically significant differences in age and clinical diagnoses of the age‐ and diagnosis‐matched subsample.

### 3.1 Cognitive decline across different ATN groups

**FIGURE 1:**
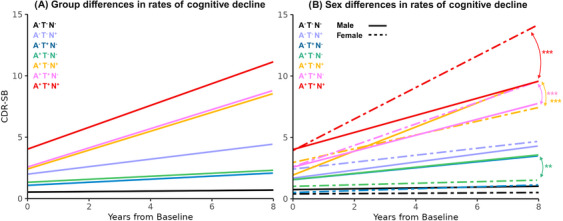
(A) Longitudinal cognitive decline trajectories across ATN groups. A, amyloid; T, tau; N, neurodegeneration; CDR‐SB, Clinical Dementia Rating–Sum of Boxes. (B) Sex differences in longitudinal cognitive decline trajectories across ATN groups. Arrows indicate ATN groups that showed significant slope differences between males and females. ** *p* < 0.005. *** *p* < 0.0001.

Figure 1A and Table S2 summarize the estimated effects of ATN pathology on cognitive trajectories as measured by CDR‐SB across time (Model 1). There was no significant impact of age at baseline, whereas education showed a small but significant impact (𝛽 = –0.1, *p* = 0.001). Except for the A^−^ T^+^ N^−^ group, all other included positive ATN pathology groups showed significantly higher baseline CDR‐SB scores compared to the control group (A^−^ T^−^ N^−^). Differences were smaller for the A^+^ T^−^ N^−^ (𝛽 = 0.76, *p* = 0.02), followed by A^+^ T^−^ N^+^ (𝛽 = 1.87, *p* < 0.001), A^+^ T^+^ N^−^ (𝛽 =  2.03, *p *< 0.001), and A^+^ T^+^ N^+^ (𝛽 = 3.50, *p* < 0.001). Similarly, all pathology‐positive groups showed faster rates of cognitive decline compared to the reference A^−^ T^−^ N^−^. As expected, participants with only one pathology present showed less steep slopes of decline (A^+^ T^−^ N^−^: 0.1 increase per year, A^−^ T^+^ N^−^: 0.1 increase per year, and A^−^ T^−^ N^+^: 0.28 increase per year; all *p* values < 0.005), whereas groups with multiple pathologies present showed even faster progression (A^+^ T^−^ N^+^: 0.75 increase per year, A^+^ T^+^ N^−^: 0.76 increase per year, and A^+^ T^+^ N^+^: 0.87 increase per year; all *p* values < 0.00001).

**FIGURE 2:**
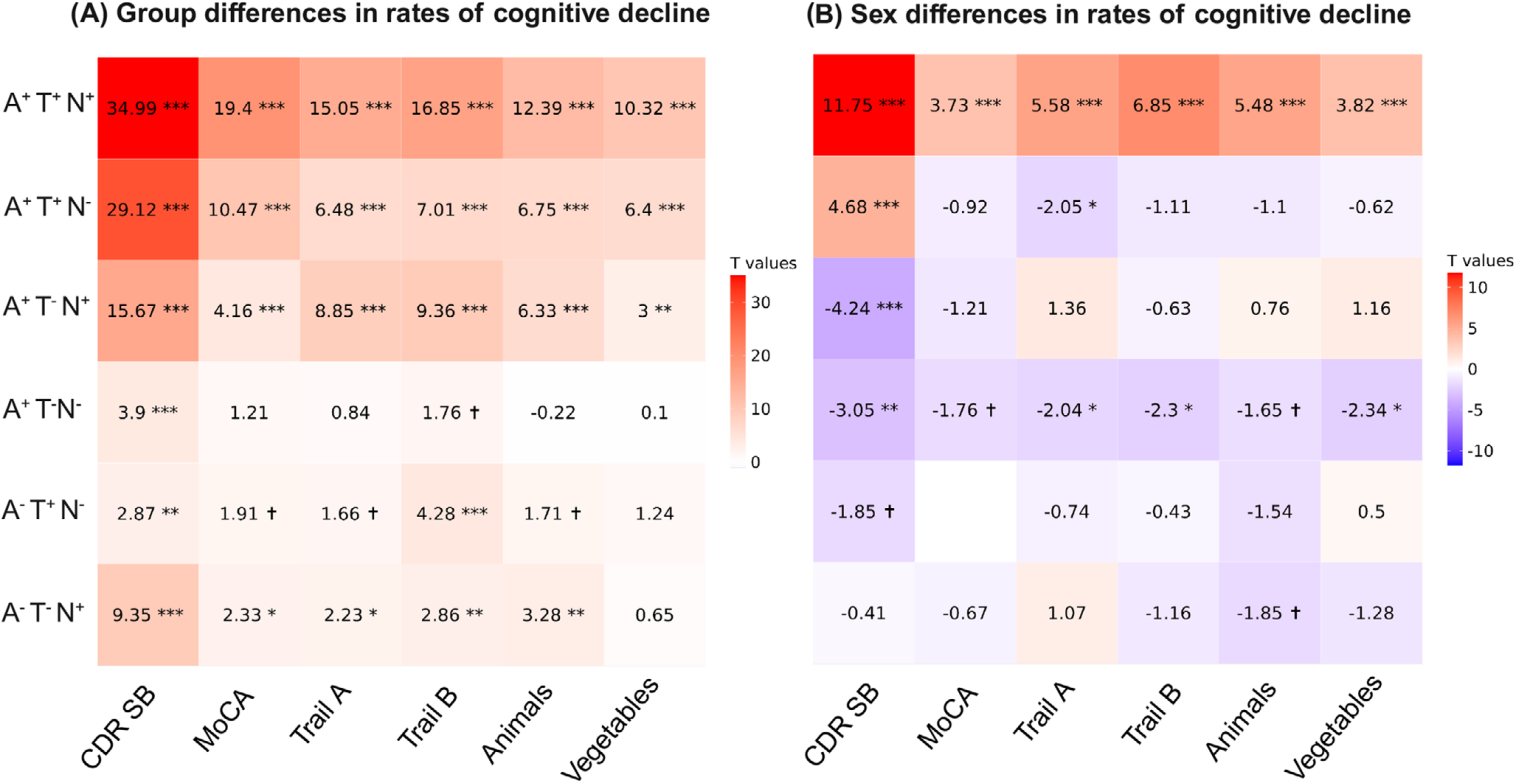
(A) Slopes of longitudinal cognitive decline trajectories across ATN groups. (B) Sex differences in longitudinal cognitive decline trajectories across ATN groups. Positive values indicate higher rates of decline in females, whereas negative values indicate higher rates of decline in males. **Note that** estimates for MoCA, Animals, and Vegetables were multiplied by (–1) so that positive estimates indicate worse cognitive performance for all tests. A, amyloid; T, tau. N, neurodegeneration; CDR‐SB, Clinical Dementia Rating–Sum of Boxes; MoCA, Montreal Cognitive Assessment. Arrows indicate ATN groups that showed significant slope differences between males and females. † *p* < 0.1; ** *p* < 0.005; *** *p* < 0.0001.

Similar patterns emerged when assessing the ATN group differences in longitudinal trajectories of MoCA, TMT A and B, and Animals, and Vegetables scores. Figure 2A shows the t‐statistics for the estimated slopes (reflecting the rates of cognitive decline compared to the reference A^−^ T^−^ N^−^ group) that were statistically significant for each group and test score. Note that estimates for MoCA, Animals, and Vegetables were multiplied by (–1) so that positive estimates indicate worse cognitive performance for all tests. Pathology‐positive groups showed faster rates of cognitive decline compared to the reference A^−^ T^−^ N^−^ group, and the A^+^ T^+^ N^+^ group had the fastest rate of cognitive decline for all tests, followed by the two pathology A^+^ T^+^ N^−^ and A^+^ T^−^ N^+^ groups. Tables S3–S7 summarize the results of these analyses.

### 3.2 Sex differences in rates of cognitive decline across ATN groups

Figure 1B and Table S8 summarize the estimated sex differences in the rates of cognitive decline across the ANT groups (Model 2). There were no significant sex differences in baseline CDR‐SB scores across ATN groups. Although there were no significant sex differences in the rates of cognitive decline in any of the A^−^ groups, we found a dichotomy based on tau status in A^+^ groups, with the A^+^ T^−^ N^−^ males showing an additional 0.17 increase in CDR‐SB score per year compared to A^+^ T^−^ N^−^ females, and the A^+^ T^−^ N^+^ males showing an additional 0.40 in CDR‐SB score per year compared to A^+^ T^−^ N^+^ females (*p* values < 0.005). Further still, female participants showed faster progression compared to males in both A^+^ T^+^ groups, with the A^+^ T^+^ N^−^ females showing an additional 0.24 increase in CDR‐SB score per year compared to A^+^ T^+^ N^−^ males, and the A^+^ T^+^ N^+^ females showing an additional 0.61 in CDR‐SB score per year compared to A^+^ T^+^ N^+^ males (*p* values < 0.00001). Repeating the analyses in the age‐ and diagnosis‐matched subsample of the data yielded similar results in terms of significance and direction of the estimated effects (Table S3).

Similar patterns of sex differences emerged when assessing the ATN group differences in the longitudinal trajectories of MoCA, TMT A and B, and Animals, and Vegetables scores. Figure 2B shows the t‐statistics estimates for the differences in the rates of cognitive decline between the sexes, contrasting the females versus males. Positive values indicate greater rates of decline in females, and negative values indicate greater rates of decline in males. Similar to CDR‐SB findings, A^+^ T^+^ N^+^ females had higher rates of cognitive decline compared to A^+^ T^+^ N^+^ males, and A^−^ T^−^ N^−^ males had higher rates of cognitive decline compared to A^−^ T^−^ N^−^ females for all tests (Rows 1 and 4 in Figure 2B). Although not statistically significant in most cases, the presence of only T^+^ or N^+^ resulted in a mixture of sex‐difference directionalities across cognitive tests, which might indicate greater levels of variability when participants are in a transitional state between A^+^ T^−^ N^−^ and A^+^ T^+^ N^+^. Tables S9–S13 summarize the results of these analyses.

### 3.3 The impact of amyloid on cognitive decline is mediated via tau and moderated by sex

**FIGURE 3:**
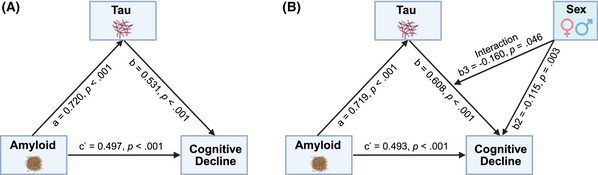
Sex differences in the mediating effect of tau positivity on the association between amyloid positivity and cognitive decline. (A) Tau mediates the relationship between amyloid and cognitive decline. (B) Sex moderates the mediation relationship between amyloid, tau, and cognitive decline. Cognitive decline indicates the rate of change in Clinical Dementia Rating–Sum of Boxes (CDR‐SB) scores.

Mediation analyses confirmed that tau positivity (T) mediated the association between amyloid positivity (A) and the rate of cognitive decline, indexed by the rate of increase in CDR‐SB score (Figure 3A), while controlling for age at baseline and education. A was significantly associated with T (a = 0.720, *z* = 40.1, *p* < 0.001). In turn, T was positively associated with the rate of CDR‐SB score increase (b = 0.531, *z* = 9.02, *p* < 0.001). The direct effect of A on the rate of cognitive decline remained significant after accounting for the indirect path through T (c′ = 0.497, *z* = 9.22, *p* < 0.001), indicating a partial mediation. The indirect effect of A on the rate of cognitive decline via T was statistically significant (estimate = 0.382, 95% CI: 0.298–0.476, *p* < 0.001), as was the direct effect (0.497, 95% CI: 0.391–0.602, *p* < 0.001). The total effect of A on CDR‐SB score change was 0.879 (95% CI: 0.798–0.958), with ≈43.5% of the effect mediated by T (95% CI: 34.1–53.9%).

Furthermore, we found a marginal moderating effect of sex on this mediation, where females showed a stronger mediation effect compared to males (Figure 3B). The main pattern of associations remained consistent, with a significant association between A and T (a = 0.719, *z* = 39.4, *p* < 0.001), T and the rate of CDR‐SB increase (b = 0.608, *z* = 8.56, *p* < 0.001), as well as a direct effect of A on the rate of CDR‐SB score increase after accounting for the indirect path through T (c′ = 0.493, *z* = 9.32, *p* < 0.001). Sex had a significant main effect on the rate of cognitive decline (b_2_ = ‐0.115, *z* = ‐2.96, *p* = 0.003), with males showing a slower decline. The interaction between tau status and sex suggested a trend toward a weaker effect of tau on decline among males (b_3_ = ‐0.160, *z* = ‐2.03, *p* = 0.045). The indirect effect of A on rate of CDR‐SB increase was significant in both sexes, but larger in females (0.437, 95% CI: 0.336–0.544, *p* < 0.001) than in males (0.322, 95% CI: 0.222–0.422, *p* < 0.001), with the proportion of the total effect mediated by T being 47.0% in females (95% CI: 37.2–56.5%) compared to 39.5% in males (95% CI: 28.1–50.5%). The difference in indirect effects between sexes approached statistical significance (0.115, 95% CI: –0.008 to 0.229, *p* = 0.046), suggesting a possible moderating effect of sex on the mediation pathway.

### 3.4 Sex differences in post‐mortem amyloid and tau burden

Adjusting for age at time of death, as expected, T^+^ participants had significantly higher postmortem Braak tangle score (𝛽 = 2.35, *p* < 0.0001), and A^+^ participants had significantly higher postmortem amyloid burden as measured by both Thal phase (𝛽 = 1.23, *p* = 0.02) and CERAD neuritic plaque score (𝛽 = 3.07, *p* < 0.0001). There were no significant sex differences in post mortem amyloid and tau burden across any of the A, T, or N groups (all *p* values > 0.05).

## 4 DISCUSSION

This study examined the longitudinal cognitive decline trajectories of aging individuals on the spectrum of AD according to their ATN classifications, and whether there were any sex differences in these trajectories within each ATN group. Our findings showed differences in rates of cognitive decline across ATN groups, whereby groups that were positive on multiple biomarkers showed steeper decline. Our main finding revealed a specific dichotomic sex difference between tau‐positive versus tau‐negative biomarker groups that had positive amyloid biomarker status. Specifically, in the A^+^T^−^ group, males showed faster cognitive decline than females in contrast to the A^+^T^+^ group, in which females showed steeper cognitive decline. These results suggest differences in the vulnerability to tau pathology in participants with positive amyloid biomarkers. This may have important clinical implications for the development of sex‐specific treatments and emphasize the importance of studying sex differences in AD.

Although no sex differences were observed in the A^−^ groups, significant sex differences were found in the A^+^ groups. Specifically, males showed steeper longitudinal cognitive decline than females in the A^+^T^−^ group. In contrast, A^+^T^+^ females showed faster progression compared to A^+^T^+^ males. Our results further confirm the presence of a mediating effect of tau pathology on the associations between amyloid positivity and cognitive decline, and suggest that these associations might differ between the sexes. Postmortem studies have identified sex differences in AD tau pathology, with a higher burden in females,^33^, ^34^ which might explain the faster progression of cognitive decline in A^+^T^+^ females. In addition, a large post‐mortem study of 1453 participants from the Religious Orders Study and the Rush Memory and Aging Project reported both higher tau and marginally higher amyloid burden in females,^35^ adjusting for age at the time of death. To test this hypothesis, we investigated the subset of participants who had post‐mortem amyloid and tau burden information available (7.5% of the sample) and did not find higher pathology burden in females or any other differences in amyloid or tau burden. This might be due to the smaller sample size with available pathological data or a ceiling effect within the tau‐positive group, with most participants having neurofibrillary Braak stage V and VI. Alternatively, several other co‐pathologies, including possible comorbid Lewy body or vascular disease not clinically detected, might have an effect on observed sex differences. Although the mechanism underlying increased rates of cognitive decline for the male participants in the A^+^T^−^ group is not clear, this may suggest a higher cognitive reserve in females than in males in earlier disease stages with a positive amyloid biomarker. Accordingly, previous studies reported a higher cognitive reserve in females.^17^, ^36^, ^37^

As expected, all N^+^ groups had greater rates of cognitive decline compared to their corresponding N^−^ groups. With a 1.49 increase in CDR‐SB score per year, the A^+^T^+^N^+^ female group had the highest rate of decline among all groups. Furthermore, the slope difference between A^+^T^+^N^+^ and A^+^T^+^N^−^ groups was greater in female participants (0.36 additional increase in CDR‐SB) compared to male participants (0.045 additional increase in CDR‐SB). This is in line with the previous literature showing stronger relationships between neurodegeneration and cognitive decline in females compared to males,^38^, ^39^ and adds to it by suggesting that this decline is further exacerbated in females in the presence of amyloid and tau.

Similar patterns of difference in the rates of cognitive decline (Figure 2A) were observed when examining other cognitive tests (MoCA, TMT A and B, Animals, and Vegetables), suggesting that the observed patterns are multi‐domain and not limited to global cognitive and functional decline. The observed sex differences in the rates of cognitive decline were also consistent with the CDR‐SB results, in particular for A^+^ T^+^N^+^ and A^+^ T^−^N^−^ groups, which showed the same dichotomy pattern (i.e., faster decline for T^+^ females and faster decline for T^−^ males; Figure 2B). The results for A^+^T^+^N^−^ and A^+^T^−^N^+^ groups were not statistically significant, likely due to the increased variability in the transition between the A^+^T^−^ N^−^ and A^+^ T^+^ N^+^ states, which might result in more heterogeneous trajectory patterns depending on the cognitive domain examined. As such, future studies with larger sample sizes are warranted to replicate these results.

Our findings have important implications for disease diagnosis and management. Sex differences in the relationships between the presence of amyloid and tau pathologies and cognitive decline might translate into sex differences in treatment efficacy when individuals receive amyloid‐clearing therapies. Because these treatments generally target patients in the earlier disease stages, who would be more likely to be A^+^T^−^, removal of amyloid might prevent cognitive decline more effectively in males than in females. This is also in line with the current evidence from recent clinical trials, where male participants benefited more from the treatment compared to their female counterparts.^8^, ^9^ Similarly, this may suggest that tau‐targeting agents^40^ might be more effective for female participants.

Although prior literature has investigated the sex differences in postmortem pathology burden along the AD continuum,^41^ as well as the sex differences in the associations between cognition and in vivo fluid (CSF or plasma) or PET amyloid and tau levels or measures of neurodegeneration,^38^, ^42^, ^43^, ^44^, ^45^ to our knowledge, differences in longitudinal cognitive decline trajectories between ATN groups has not been investigated, as is the focus of the current study. Our study has been enabled by the large sample size of participants with PET and CSF amyloid and tau biomarkers and longitudinal assessments provided by the NACC (1464 participants, 7168 longitudinal assessments, 4.77 ± 3.78 years of follow‐up), allowing us to robustly model the potential differences across ATN groups.

Although our study includes a large sample size with considerable longitudinal assessments, allowing for robust estimation of longitudinal cognitive trajectories across ATN groups, we acknowledge certain limitations. First, amyloid and tau positivity were defined based on either PET or CSF assessments, leading to potential variability in the measurements. However, the literature suggests strong agreements between PET and CSF based assessments for both amyloid and tau pathologies.^46^, ^47^, ^48^ In addition, we found similarly high levels of agreement (88.2% for amyloid status and 87.4% for tau status) in the subsets of participants who had both CSF and PET data available, suggesting that the impact of using either biomarker on the results would be minimal in the context of this study. In addition, sex differences were found in the demographic characteristics of the included participants, whereby the female participants were younger, and the sample included a higher proportion of female participants that were cognitively normal at baseline. To control for these differences, the sex difference analyses (Model 2) were repeated in an age‐ and diagnosis‐matched subset of the data, and the obtained results remained similar. Finally, due to data availability and sample size limitations, we were not able to incorporate vascular pathology (V) in the analyses. Because vascular pathology is a common comorbidity that exacerbates cognitive decline in aging individuals as well as patients with AD^49^, ^50^, ^51^, ^52^, ^53^ and previous studies have reported sex differences in prevalence and impact of vascular pathology on cognitive decline trajectories,^54^, ^55^ future studies assessing sex differences in cognitive decline trajectories across ATN‐V groups are warranted.

In conclusion, we identified a distinct sex‐specific dichotomy in cognitive decline trajectories among individuals with amyloid‐positive biomarkers. Specifically, males exhibited a more rapid cognitive decline than females in the absence of tau pathology, whereas females experienced a steeper decline when tau biomarkers were also present. These findings may hold significant clinical implications for the development of sex‐specific therapeutic strategies for AD.

## AUTHOR CONTRIBUTIONS

All authors were involved with design, conceptualization, and interpretation of the findings. Yashar Zeighami and Mahsa Dadar completed the analyses and drafted the manuscript. All authors revised and approved the submitted version.

## CONFLICT OF INTEREST STATEMENT

The authors declare no competing interests. Author disclosures are available in the supporting information.

## DISCLOSURES

The authors report no disclosures relevant to the manuscript.

## CONSENT STATEMENT

Written informed consent was obtained from participants or their study partners.

## Supporting information



Supporting Information

Supporting Information

## Data Availability

Data used in preparation of this article were obtained from the National Alzheimer's Coordinating Center (NACC; https://naccdata.org/) database.
