# Supplementary material for: Sex differences in vulnerability to tau pathology: Impact on cognitive decline
Source: Alzheimers Dement. 2025 Oct 8;21(10):e70634. doi: 10.1002/alz.70634 (PMC12505017; doi:10.1002/alz.70634)
Supplement: Supplementary file 1 — Supporting Information [file ALZ-21-e70634-s001.docx]

**Supplementary Table 1.** Names and descriptions of NACC variables included in the present study.

| Term used | Variable Name | Description |
| --- | --- | --- |
| PET-based Amyloid positivity | AMYLPET | Amyloid positivity status based on amyloid PET |
| CSF-based Amyloid positivity | AMYLCSF | Amyloid positivity status based on CSF |
| PET-based Tau positivity | TAUPETAD | Tau positivity status based on amyloid PET |
| CSF-based Tau positivity | CSFTAU | Tau positivity status based on CSF |
| Hippocampal atrophy | HIPPATR | Hippocampal atrophy status based on MRI |
| CDR sum of boxes | CDRSUM | Clinical dementia rating sum of boxes (CDR-SB) |
| MoCA | NACCMOCA | MoCA Total Score — corrected for education |
| Trail A | TRAILA | Trail Making Test Part A — Total number of seconds to complete |
| Trail B | TRAILB | Trail Making Test Part B — Total number of seconds to complete |
| Animals | ANIMALS | Animals — Total number of animals named in 60 seconds |
| Vegetables | VEG | Vegetable — Total number of vegetables named in 60 seconds |
| Clinical diagnosis | NACCETPR | Clinical diagnosis |
| Thal amyloid phase | NPTHAL | Thal amyloid phase |
| CERAD amyloid score | NACCNEUR | CERAD amyloid score |
| Braak tau stage | NACCBRAA | Braak tau stage |

PET: Positron emission tomography. CSF: Cerebrospinal fluid. CERAD: Consortium to Establish a Registry for Alzheimer’s Disease. MoCA: Montreal Cognitive Assessment.

**Supplementary Table 2.** Model 1 estimates: **CDR-SB ~** **Time from Baseline : ATN** + Time from Baseline + ATN + Age_bl_ + Education + (1|ID)

| Parameter | Coefficient | SE | CI_low | CI_high | T stat | P value |
| --- | --- | --- | --- | --- | --- | --- |
| (Intercept) | 3.381 | 0.835 | 1.745 | 5.017 | 4.051 | <0.0001 |
| Age_bl_ | -0.018 | 0.011 | -0.039 | 0.003 | -1.718 | 0.085 |
| Time from Baseline | 0.021 | 0.009 | 0.004 | 0.038 | 2.381 | 0.017 |
| A^-^T^-^N^+^ | 1.421 | 0.334 | 0.766 | 2.077 | 4.25 | <0.0001 |
| A^-^T^+^N^-^ | 0.356 | 0.495 | -0.614 | 1.326 | 0.719 | 0.471 |
| A1T^-^N^-^ | 0.756 | 0.324 | 0.121 | 1.391 | 2.332 | 0.019 |
| A^+^T^-^N^+^ | 1.865 | 0.457 | 0.969 | 2.762 | 4.08 | <0.0001 |
| A^+^T^+^N^-^ | 2.03 | 0.244 | 1.551 | 2.509 | 8.308 | <0.0001 |
| A^+^T^+^N^+^ | 3.503 | 0.215 | 3.081 | 3.925 | 16.273 | <0.0001 |
| Education | -0.098 | 0.03 | -0.158 | -0.039 | -3.266 | 0.001 |
| Time from Baseline:A^-^T^-^N^+^ | 0.284 | 0.03 | 0.224 | 0.344 | 9.349 | <0.0001 |
| Time from Baseline:A^-^T^+^N^-^ | 0.105 | 0.036 | 0.033 | 0.176 | 2.874 | 0.004 |
| Time from Baseline:A^+^T^-^N^-^ | 0.102 | 0.026 | 0.05 | 0.153 | 3.9 | <0.0001 |
| Time from Baseline:A^+^T^-^N^+^ | 0.747 | 0.048 | 0.653 | 0.84 | 15.671 | <0.0001 |
| Time from Baseline:A^+^T^+^N^-^ | 0.758 | 0.026 | 0.707 | 0.809 | 29.124 | <0.0001 |
| Time from Baseline:A^+^T^+^N^+^ | 0.867 | 0.025 | 0.818 | 0.916 | 34.991 | <0.0001 |

ATN: Categorical variable determining amyloid, tau, and neurodegeneration positivity status. Age_bl_ : Age at baseline. ID: Categorical variable indicating participant ID. CDR-SB: Clinical dementia rating sum of boxes.

**Supplementary Table 3.** Model 1 estimates: **MoCA ~** **Time from Baseline : ATN** + Time from Baseline + ATN + Age_bl_ + Education + (1|ID)

| Parameter | Coefficient | SE | CI_low | CI_high | T stat | P value |
| --- | --- | --- | --- | --- | --- | --- |
| (Intercept) | −0.427 | 0.211 | −0.84 | −0.014 | −2.028 | 0.04258 |
| Age_bl_ | 0 | 0.003 | −0.006 | 0.006 | −0.048 | 0.96175 |
| Time from Baseline | −0.004 | 0.004 | −0.011 | 0.003 | −1.143 | 0.25331 |
| A^-^T^-^N^+^ | 0.383 | 0.109 | 0.17 | 0.596 | 3.528 | 0.00042 |
| A^-^T^+^N^-^ | 0.07 | 0.136 | −0.198 | 0.337 | 0.512 | 0.6084 |
| A1T^-^N^-^ | 0.193 | 0.093 | 0.01 | 0.376 | 2.068 | 0.03871 |
| A^+^T^-^N^+^ | 0.871 | 0.142 | 0.592 | 1.15 | 6.115 | <0.0001 |
| A^+^T^+^N^-^ | 0.807 | 0.072 | 0.666 | 0.949 | 11.177 | <0.0001 |
| A^+^T^+^N^+^ | 1.304 | 0.064 | 1.178 | 1.43 | 20.287 | <0.0001 |
| Education | −0.17 | 0.023 | −0.215 | −0.126 | −7.541 | <0.0001 |
| Time from Baseline:A^-^T^-^N^+^ | 0.032 | 0.014 | 0.005 | 0.058 | 2.329 | 0.01993 |
| Time from Baseline:A^-^T^+^N^-^ | 0.026 | 0.014 | −0.001 | 0.053 | 1.912 | 0.05597 |
| Time from Baseline:A^+^T^-^N^-^ | 0.013 | 0.011 | −0.008 | 0.033 | 1.213 | 0.22503 |
| Time from Baseline:A^+^T^-^N^+^ | 0.077 | 0.018 | 0.041 | 0.113 | 4.164 | <0.0001 |
| Time from Baseline:A^+^T^+^N^-^ | 0.118 | 0.011 | 0.096 | 0.14 | 10.469 | <0.0001 |
| Time from Baseline:A^+^T^+^N^+^ | 0.184 | 0.01 | 0.166 | 0.203 | 19.396 | <0.0001 |

ATN: Categorical variable determining amyloid, tau, and neurodegeneration positivity status. Age_bl_ : Age at baseline. ID: Categorical variable indicating participant ID. MoCA: Montreal Cognitive Assessment.

**Supplementary Table 4.** Model 1 estimates: **Trail-A ~** **Time from Baseline : ATN** + Time from Baseline + ATN + Age_bl_ + Education + (1|ID)

| Parameter | Coefficient | SE | CI_low | CI_high | T stat | P value |
| --- | --- | --- | --- | --- | --- | --- |
| (Intercept) | −0.732 | 0.244 | −1.211 | −0.253 | −2.993 | 0.00277 |
| Age_bl_ | 0.007 | 0.004 | 0 | 0.014 | 2.004 | 0.04512 |
| Time from Baseline | 0.007 | 0.003 | 0.001 | 0.014 | 2.386 | 0.01707 |
| A^-^T^-^N^+^ | 0.233 | 0.114 | 0.009 | 0.457 | 2.035 | 0.04191 |
| A^-^T^+^N^-^ | −0.012 | 0.156 | −0.317 | 0.293 | −0.078 | 0.93811 |
| A1T^-^N^-^ | 0.218 | 0.106 | 0.01 | 0.427 | 2.055 | 0.03995 |
| A^+^T^-^N^+^ | 0.498 | 0.151 | 0.201 | 0.794 | 3.289 | 0.00101 |
| A^+^T^+^N^-^ | 0.597 | 0.085 | 0.431 | 0.763 | 7.045 | <0.0001 |
| A^+^T^+^N^+^ | 0.88 | 0.075 | 0.732 | 1.027 | 11.694 | <0.0001 |
| Education | −0.153 | 0.026 | −0.204 | −0.101 | −5.803 | <0.0001 |
| Time from Baseline:A^-^T^-^N^+^ | 0.026 | 0.012 | 0.003 | 0.049 | 2.228 | 0.02591 |
| Time from Baseline:A^-^T^+^N^-^ | 0.022 | 0.013 | −0.004 | 0.047 | 1.658 | 0.09743 |
| Time from Baseline:A^+^T^-^N^-^ | 0.008 | 0.01 | −0.011 | 0.028 | 0.836 | 0.40333 |
| Time from Baseline:A^+^T^-^N^+^ | 0.161 | 0.018 | 0.125 | 0.196 | 8.853 | <0.0001 |
| Time from Baseline:A^+^T^+^N^-^ | 0.078 | 0.012 | 0.054 | 0.102 | 6.481 | <0.0001 |
| Time from Baseline:A^+^T^+^N^+^ | 0.145 | 0.01 | 0.126 | 0.164 | 15.05 | <0.0001 |

ATN: Categorical variable determining amyloid, tau, and neurodegeneration positivity status. Age_bl_ : Age at baseline. ID: Categorical variable indicating participant ID. Trail-A: Trail making test part A.

**Supplementary Table 5.** Model 1 estimates: **Trail-B ~** **Time from Baseline : ATN** + Time from Baseline + ATN + Age_bl_ + Education + (1|ID)

| Parameter | Coefficient | SE | CI_low | CI_high | T stat | P value |
| --- | --- | --- | --- | --- | --- | --- |
| (Intercept) | −1.805 | 0.237 | −2.27 | −1.341 | −7.62 | <0.0001 |
| Age_bl_ | 0.023 | 0.003 | 0.016 | 0.03 | 6.537 | <0.0001 |
| Time from Baseline | 0.012 | 0.003 | 0.006 | 0.018 | 3.9 | <0.0001 |
| A^-^T^-^N^+^ | 0.432 | 0.106 | 0.224 | 0.64 | 4.077 | <0.0001 |
| A^-^T^+^N^-^ | −0.01 | 0.144 | −0.292 | 0.271 | −0.071 | 0.94352 |
| A1T^-^N^-^ | 0.049 | 0.102 | −0.151 | 0.25 | 0.482 | 0.62974 |
| A^+^T^-^N^+^ | 0.665 | 0.145 | 0.381 | 0.949 | 4.589 | <0.0001 |
| A^+^T^+^N^-^ | 0.53 | 0.083 | 0.368 | 0.693 | 6.404 | <0.0001 |
| A^+^T^+^N^+^ | 0.936 | 0.075 | 0.789 | 1.084 | 12.418 | <0.0001 |
| Education | −0.172 | 0.025 | −0.222 | −0.123 | −6.785 | <0.0001 |
| Time from Baseline:A^-^T^-^N^+^ | 0.034 | 0.012 | 0.011 | 0.057 | 2.862 | 0.00423 |
| Time from Baseline:A^-^T^+^N^-^ | 0.054 | 0.013 | 0.029 | 0.079 | 4.278 | <0.0001 |
| Time from Baseline:A^+^T^-^N^-^ | 0.017 | 0.01 | −0.002 | 0.036 | 1.763 | 0.078 |
| Time from Baseline:A^+^T^-^N^+^ | 0.183 | 0.02 | 0.145 | 0.222 | 9.363 | <0.0001 |
| Time from Baseline:A^+^T^+^N^-^ | 0.084 | 0.012 | 0.061 | 0.108 | 7.014 | <0.0001 |
| Time from Baseline:A^+^T^+^N^+^ | 0.167 | 0.01 | 0.147 | 0.186 | 16.848 | <0.0001 |

ATN: Categorical variable determining amyloid, tau, and neurodegeneration positivity status. Age_bl_ : Age at baseline. ID: Categorical variable indicating participant ID. Trail-B: Trail making test part B.

**Supplementary Table 6.** Model 1 estimates: **Animals ~** **Time from Baseline : ATN** + Time from Baseline + ATN + Age_bl_ + Education + (1|ID)

| Parameter | Coefficient | SE | CI_low | CI_high | T stat | P value |
| --- | --- | --- | --- | --- | --- | --- |
| (Intercept) | −1.352 | 0.188 | −1.721 | −0.982 | −7.177 | <0.0001 |
| Age_bl_ | 0.015 | 0.003 | 0.01 | 0.02 | 5.435 | <0.0001 |
| Time from Baseline | 0.015 | 0.003 | 0.009 | 0.02 | 5.553 | <0.0001 |
| A^-^T^-^N^+^ | 0.413 | 0.089 | 0.239 | 0.587 | 4.643 | <0.0001 |
| A^-^T^+^N^-^ | 0.18 | 0.125 | −0.066 | 0.426 | 1.438 | 0.15062 |
| A1T^-^N^-^ | 0.177 | 0.084 | 0.013 | 0.341 | 2.119 | 0.03411 |
| A^+^T^-^N^+^ | 0.583 | 0.12 | 0.349 | 0.817 | 4.877 | <0.0001 |
| A^+^T^+^N^-^ | 0.724 | 0.065 | 0.596 | 0.851 | 11.132 | <0.0001 |
| A^+^T^+^N^+^ | 1.159 | 0.057 | 1.047 | 1.272 | 20.181 | <0.0001 |
| Education | −0.182 | 0.02 | −0.222 | −0.143 | −9.009 | <0.0001 |
| Time from Baseline:A^-^T^-^N^+^ | 0.031 | 0.009 | 0.012 | 0.049 | 3.275 | 0.00106 |
| Time from Baseline:A^-^T^+^N^-^ | 0.019 | 0.011 | −0.003 | 0.04 | 1.715 | 0.08646 |
| Time from Baseline:A^+^T^-^N^-^ | −0.002 | 0.008 | −0.018 | 0.014 | −0.217 | 0.82834 |
| Time from Baseline:A^+^T^-^N^+^ | 0.1 | 0.016 | 0.069 | 0.131 | 6.327 | <0.0001 |
| Time from Baseline:A^+^T^+^N^-^ | 0.067 | 0.01 | 0.048 | 0.086 | 6.754 | <0.0001 |
| Time from Baseline:A^+^T^+^N^+^ | 0.1 | 0.008 | 0.084 | 0.116 | 12.39 | <0.0001 |

ATN: Categorical variable determining amyloid, tau, and neurodegeneration positivity status. Age_bl_ : Age at baseline. ID: Categorical variable indicating participant ID.

**Supplementary Table 7.** Model 1 estimates: **Vegetables ~** **Time from Baseline : ATN** + Time from Baseline + ATN + Age_bl_ + Education + (1|ID)

| Parameter | Coefficient | SE | CI_low | CI_high | T stat | P value |
| --- | --- | --- | --- | --- | --- | --- |
| (Intercept) | −1.394 | 0.185 | −1.756 | −1.031 | −7.541 | <0.0001 |
| Age_bl_ | 0.015 | 0.003 | 0.009 | 0.02 | 5.384 | <0.0001 |
| Time from Baseline | 0.024 | 0.003 | 0.019 | 0.029 | 8.796 | <0.0001 |
| A^-^T^-^N^+^ | 0.631 | 0.087 | 0.46 | 0.802 | 7.232 | <0.0001 |
| A^-^T^+^N^-^ | 0.211 | 0.123 | −0.03 | 0.452 | 1.718 | 0.08581 |
| A1T^-^N^-^ | 0.253 | 0.082 | 0.092 | 0.415 | 3.077 | 0.0021 |
| A^+^T^-^N^+^ | 0.882 | 0.117 | 0.652 | 1.112 | 7.519 | <0.0001 |
| A^+^T^+^N^-^ | 0.851 | 0.064 | 0.726 | 0.977 | 13.291 | <0.0001 |
| A^+^T^+^N^+^ | 1.254 | 0.057 | 1.143 | 1.365 | 22.054 | <0.0001 |
| Education | −0.096 | 0.02 | −0.135 | −0.057 | −4.85 | <0.0001 |
| Time from Baseline:A^-^T^-^N^+^ | 0.006 | 0.01 | −0.013 | 0.026 | 0.653 | 0.51359 |
| Time from Baseline:A^-^T^+^N^-^ | 0.014 | 0.011 | −0.008 | 0.036 | 1.242 | 0.21438 |
| Time from Baseline:A^+^T^-^N^-^ | 0.001 | 0.008 | −0.016 | 0.017 | 0.1 | 0.92036 |
| Time from Baseline:A^+^T^-^N^+^ | 0.049 | 0.016 | 0.017 | 0.081 | 2.997 | 0.00274 |
| Time from Baseline:A^+^T^+^N^-^ | 0.066 | 0.01 | 0.046 | 0.086 | 6.396 | <0.0001 |
| Time from Baseline:A^+^T^+^N^+^ | 0.086 | 0.008 | 0.07 | 0.103 | 10.32 | <0.0001 |

ATN: Categorical variable determining amyloid, tau, and neurodegeneration positivity status. Age_bl_ : Age at baseline. ID: Categorical variable indicating participant ID.

**Supplementary Table 8.** Model 2 estimates: **CDR-SB ~** **Time from Baseline : ATN : Sex** + Time from Baseline : ATN + Time from Baseline + ATN + Time from Baseline : Sex + ATN : Sex + Sex + Age_bl_ + Education + (1|ID)

| Parameter | Coefficient | SE | CI_low | CI_high | T stat | P value |
| --- | --- | --- | --- | --- | --- | --- |
| (Intercept) | 3.484 | 0.874 | 1.772 | 5.197 | 3.989 | <0.0001 |
| Age_bl_ | -0.018 | 0.011 | -0.039 | 0.003 | -1.713 | 0.086 |
| Time from Baseline | 0.034 | 0.014 | 0.006 | 0.062 | 2.363 | 0.018 |
| A^-^T^-^N^+^ | 0.934 | 0.434 | 0.084 | 1.785 | 2.154 | 0.031 |
| A^-^T^+^N^-^ | 0.808 | 0.83 | -0.819 | 2.436 | 0.973 | 0.330 |
| A^+^T^-^N^-^ | 0.821 | 0.493 | -0.146 | 1.787 | 1.664 | 0.096 |
| A^+^T^-^N^+^ | 1.184 | 0.593 | 0.022 | 2.346 | 1.997 | 0.045 |
| A^+^T^+^N^-^ | 1.825 | 0.359 | 1.12 | 2.53 | 5.077 | <0.0001 |
| A^+^T^+^N^+^ | 3.265 | 0.308 | 2.661 | 3.868 | 10.608 | <0.0001 |
| Sex_Female_ | -0.349 | 0.268 | -0.875 | 0.177 | -1.3 | 0.193 |
| Education | -0.092 | 0.03 | -0.151 | -0.033 | -3.032 | 0.002 |
| Time from Baseline:A^-^T^-^N^+^ | 0.293 | 0.044 | 0.207 | 0.379 | 6.683 | <0.0001 |
| Time from Baseline:A^-^T^+^N^-^ | 0.208 | 0.066 | 0.079 | 0.337 | 3.17 | 0.001 |
| Time from Baseline:A^+^T^-^N^-^ | 0.217 | 0.045 | 0.128 | 0.305 | 4.809 | <0.0001 |
| Time from Baseline:A^+^T^-^N^+^ | 0.945 | 0.067 | 0.813 | 1.076 | 14.084 | <0.0001 |
| Time from Baseline:A^+^T^+^N^-^ | 0.616 | 0.04 | 0.538 | 0.694 | 15.482 | <0.0001 |
| Time from Baseline:A^+^T^+^N^+^ | 0.661 | 0.031 | 0.599 | 0.722 | 21.031 | <0.0001 |
| Time from Baseline:Sex_Female_ | -0.02 | 0.018 | -0.056 | 0.015 | -1.136 | 0.256 |
| A^-^T^-^N^+^:Sex_Female_ | 1.085 | 0.685 | -0.257 | 2.427 | 1.585 | 0.113 |
| A^-^T^+^N^-^:Sex_Female_ | -0.696 | 1.028 | -2.71 | 1.319 | -0.677 | 0.498 |
| A^+^T^-^N^-^:Sex_Female_ | -0.206 | 0.648 | -1.477 | 1.065 | -0.318 | 0.750 |
| A^+^T^-^N^+^:Sex_Female_ | 1.372 | 0.926 | -0.443 | 3.186 | 1.482 | 0.138 |
| A^+^T^+^N^-^:Sex_Female_ | 0.378 | 0.488 | -0.578 | 1.334 | 0.775 | 0.438 |
| A^+^T^+^N^+^:Sex_Female_ | 0.265 | 0.433 | -0.584 | 1.115 | 0.613 | 0.540 |
| Time from Baseline:A^-^T^-^N^+^:Sex_Female_ | -0.025 | 0.06 | -0.143 | 0.093 | -0.411 | 0.681 |
| Time from Baseline:A^-^T^+^N^-^:Sex_Female_ | -0.145 | 0.078 | -0.298 | 0.009 | -1.846 | 0.064 |
| Time from Baseline:A^+^T^-^N^-^:Sex_Female_ | -0.167 | 0.055 | -0.275 | -0.06 | -3.053 | 0.002 |
| Time from Baseline:A^+^T^-^N^+^:Sex_Female_ | -0.399 | 0.094 | -0.583 | -0.214 | -4.243 | <0.0001 |
| Time from Baseline:A^+^T^+^N^-^:Sex_Female_ | 0.243 | 0.052 | 0.142 | 0.345 | 4.682 | <0.0001 |
| Time from Baseline:A^+^T^+^N^+^:Sex_Female_ | 0.608 | 0.052 | 0.506 | 0.709 | 11.749 | <0.0001 |

ATN: Categorical variable determining amyloid, tau, and neurodegeneration positivity status. Age_bl_ : Age at baseline. ID: Categorical variable indicating participant ID. CDR-SB: Clinical dementia rating sum of boxes.

**Supplementary Table 9.** Model 2 estimates: **MoCA ~** **Time from Baseline : ATN : Sex** + Time from Baseline : ATN + Time from Baseline + ATN + Time from Baseline : Sex + ATN : Sex + Sex + Age_bl_ + Education + (1|ID)

| Parameter | Coefficient | SE | CI_low | CI_high | T stat | P value |
| --- | --- | --- | --- | --- | --- | --- |
| (Intercept) | −18.731 | 1.592 | −21.854 | −15.609 | −11.763 | <0.0001 |
| Age_bl_ | −0.005 | 0.019 | −0.043 | 0.032 | −0.271 | 0.78616 |
| Time from Baseline | −0.038 | 0.036 | −0.109 | 0.033 | −1.038 | 0.29957 |
| A^-^T^-^N^+^ | 1.883 | 0.825 | 0.266 | 3.501 | 2.283 | 0.02246 |
| A^+^T^-^N^-^ | 2.247 | 0.88 | 0.521 | 3.973 | 2.553 | 0.01 |
| A^+^T^-^N^+^ | 4.002 | 1.153 | 1.741 | 6.263 | 3.47 | <0.0001 |
| A^+^T^+^N^-^ | 4.382 | 0.633 | 3.14 | 5.624 | 6.918 | <0.0001 |
| A^+^T^+^N^+^ | 6.815 | 0.555 | 5.727 | 7.903 | 12.279 | <0.0001 |
| Sex_Female_ | −0.941 | 0.483 | −1.887 | 0.006 | −1.947 | 0.05155 |
| Education | −0.404 | 0.055 | −0.511 | −0.296 | −7.356 | <0.0001 |
| Time from Baseline:A^-^T^-^N^+^ | 0.245 | 0.107 | 0.034 | 0.455 | 2.281 | 0.02262 |
| Time from Baseline:A^+^T^-^N^-^ | 0.242 | 0.11 | 0.027 | 0.456 | 2.207 | 0.0274 |
| Time from Baseline:A^+^T^-^N^+^ | 0.611 | 0.165 | 0.288 | 0.935 | 3.702 | <0.0001 |
| Time from Baseline:A^+^T^+^N^-^ | 0.792 | 0.099 | 0.599 | 0.985 | 8.03 | <0.0001 |
| Time from Baseline:A^+^T^+^N^+^ | 1.015 | 0.073 | 0.873 | 1.158 | 13.999 | <0.0001 |
| Time from Baseline:Sex_Female_ | 0.019 | 0.046 | −0.072 | 0.109 | 0.41 | 0.6818 |
| A^-^T^-^N^+^:Sex_Female_ | 0.608 | 1.448 | −2.231 | 3.446 | 0.42 | 0.6748 |
| A^+^T^-^N^-^:Sex_Female_ | −1.989 | 1.152 | −4.248 | 0.27 | −1.726 | 0.08438 |
| A^+^T^-^N^+^:Sex_Female_ | 2.813 | 1.76 | −0.637 | 6.263 | 1.598 | 0.11003 |
| A^+^T^+^N^-^:Sex_Female_ | 0.938 | 0.889 | −0.806 | 2.681 | 1.055 | 0.29169 |
| A^+^T^+^N^+^:Sex_Female_ | 2.346 | 0.799 | 0.779 | 3.912 | 2.935 | 0.00335 |
| Time from Baseline:A^-^T^-^N^+^:Sex_Female_ | −0.114 | 0.171 | −0.449 | 0.22 | −0.67 | 0.5028 |
| Time from Baseline:A^+^T^-^N^-^:Sex_Female_ | −0.238 | 0.135 | −0.504 | 0.027 | −1.763 | 0.07796 |
| Time from Baseline:A^+^T^-^N^+^:Sex_Female_ | −0.273 | 0.226 | −0.717 | 0.17 | −1.21 | 0.22651 |
| Time from Baseline:A^+^T^+^N^-^:Sex_Female_ | −0.127 | 0.138 | −0.398 | 0.144 | −0.917 | 0.35922 |
| Time from Baseline:A^+^T^+^N^+^:Sex_Female_ | 0.478 | 0.128 | 0.227 | 0.729 | 3.733 | 0.00019 |

ATN: Categorical variable determining amyloid, tau, and neurodegeneration positivity status. Age_bl_ : Age at baseline. ID: Categorical variable indicating participant ID. MoCA: Montreal Cognitive Assessment score.

**Supplementary Table 10.** Model 2 estimates: **Trail_A ~** **Time from Baseline : ATN : Sex** + Time from Baseline : ATN + Time from Baseline + ATN + Time from Baseline : Sex + ATN : Sex + Sex + Age_bl_ + Education + (1|ID)

| Parameter | Coefficient | SE | CI_low | CI_high | T stat | P value |
| --- | --- | --- | --- | --- | --- | --- |
| (Intercept) | 44.184 | 7.528 | 29.426 | 58.943 | 5.869 | <0.0001 |
| Age_bl_ | 0.176 | 0.091 | −0.003 | 0.355 | 1.929 | 0.05382 |
| Time from Baseline | 0.267 | 0.131 | 0.01 | 0.525 | 2.038 | 0.0417 |
| A^-^T^-^N^+^ | 3.722 | 3.765 | −3.658 | 11.102 | 0.989 | 0.323 |
| A^-^T^+^N^-^ | 0.227 | 6.571 | −12.655 | 13.108 | 0.035 | 0.97247 |
| A^+^T^-^N^-^ | 6.185 | 4.177 | −2.004 | 14.374 | 1.481 | 0.139 |
| A^+^T^-^N^+^ | 11.983 | 5.044 | 2.095 | 21.872 | 2.376 | 0.01755 |
| A^+^T^+^N^-^ | 14.568 | 3.12 | 8.452 | 20.684 | 4.67 | <0.0001 |
| A^+^T^+^N^+^ | 20.125 | 2.64 | 14.95 | 25.3 | 7.624 | <0.0001 |
| Sex_Female_ | −2.015 | 2.194 | −6.315 | 2.285 | −0.919 | 0.358 |
| Education | −1.497 | 0.26 | −2.007 | −0.987 | −5.754 | <0.0001 |
| Time from Baseline:A^-^T^-^N^+^ | 0.291 | 0.441 | −0.573 | 1.155 | 0.66 | 0.50956 |
| Time from Baseline:A^-^T^+^N^-^ | 0.917 | 0.586 | −0.232 | 2.066 | 1.564 | 0.11781 |
| Time from Baseline:A^+^T^-^N^-^ | 0.925 | 0.427 | 0.088 | 1.761 | 2.168 | 0.03023 |
| Time from Baseline:A^+^T^-^N^+^ | 3.259 | 0.739 | 1.811 | 4.708 | 4.411 | <0.0001 |
| Time from Baseline:A^+^T^+^N^-^ | 2.721 | 0.478 | 1.783 | 3.659 | 5.689 | <0.0001 |
| Time from Baseline:A^+^T^+^N^+^ | 2.946 | 0.287 | 2.383 | 3.51 | 10.251 | <0.0001 |
| Time from Baseline:Sex_Female_ | −0.122 | 0.164 | −0.442 | 0.199 | −0.742 | 0.45797 |
| A^-^T^-^N^+^:Sex_Female_ | 4.879 | 5.948 | −6.782 | 16.54 | 0.82 | 0.41211 |
| A^-^T^+^N^-^:Sex_Female_ | −0.776 | 8.18 | −16.813 | 15.26 | −0.095 | 0.92438 |
| A^+^T^-^N^-^:Sex_Female_ | −1.528 | 5.437 | −12.187 | 9.13 | −0.281 | 0.77865 |
| A^+^T^-^N^+^:Sex_Female_ | 1.342 | 7.767 | −13.885 | 16.568 | 0.173 | 0.86285 |
| A^+^T^+^N^-^:Sex_Female_ | 0.004 | 4.299 | −8.425 | 8.432 | 0.001 | 0.99933 |
| A^+^T^+^N^+^:Sex_Female_ | 2.472 | 3.896 | −5.166 | 10.109 | 0.634 | 0.52582 |
| Time from Baseline:A^-^T^-^N^+^:Sex_Female_ | 0.633 | 0.592 | −0.528 | 1.793 | 1.069 | 0.28529 |
| Time from Baseline:A^-^T^+^N^-^:Sex_Female_ | −0.526 | 0.707 | −1.911 | 0.859 | −0.745 | 0.45637 |
| Time from Baseline:A^+^T^-^N^-^:Sex_Female_ | −1.069 | 0.525 | −2.098 | −0.04 | −2.036 | 0.0418 |
| Time from Baseline:A^+^T^-^N^+^:Sex_Female_ | 1.277 | 0.939 | −0.563 | 3.117 | 1.36 | 0.17381 |
| Time from Baseline:A^+^T^+^N^-^:Sex_Female_ | −1.263 | 0.617 | −2.473 | −0.053 | −2.046 | 0.04084 |
| Time from Baseline:A^+^T^+^N^+^:Sex_Female_ | 3.309 | 0.593 | 2.146 | 4.472 | 5.579 | <0.0001 |

ATN: Categorical variable determining amyloid, tau, and neurodegeneration positivity status. Age_bl_ : Age at baseline. ID: Categorical variable indicating participant ID. Trail_A: Trail making test part A.

**Supplementary Table 11.** Model 2 estimates: **Trail_B ~** **Time from Baseline : ATN : Sex** + Time from Baseline : ATN + Time from Baseline + ATN + Time from Baseline : Sex + ATN : Sex + Sex + Age_bl_ + Education + (1|ID)

| Parameter | Coefficient | SE | CI_low | CI_high | T stat | P value |
| --- | --- | --- | --- | --- | --- | --- |
| (Intercept) | 61.576 | 19.105 | 24.122 | 99.03 | 3.223 | 0.0128 |
| Age_bl_ | 1.446 | 0.231 | 0.993 | 1.899 | 6.251 | <0.0001 |
| Time from Baseline | 0.523 | 0.328 | −0.121 | 1.166 | 1.593 | 0.11129 |
| A^-^T^-^N^+^ | 14.122 | 9.102 | −3.723 | 31.966 | 1.551 | 0.121 |
| A^-^T^+^N^-^ | −9.467 | 15.579 | −40.009 | 21.076 | −0.608 | 0.54345 |
| A^+^T^-^N^-^ | 5.669 | 10.427 | −14.772 | 26.11 | 0.544 | 0.58668 |
| A^+^T^-^N^+^ | 30.552 | 12.627 | 5.798 | 55.306 | 2.42 | 0.01557 |
| A^+^T^+^N^-^ | 25.84 | 7.929 | 10.295 | 41.385 | 3.259 | 0.0014 |
| A^+^T^+^N^+^ | 59.319 | 6.63 | 46.322 | 72.316 | 8.947 | <0.0001 |
| Sex_Female_ | −11.388 | 5.251 | −21.682 | −1.094 | −2.169 | 0.03015 |
| Education | −4.563 | 0.674 | −5.885 | −3.241 | −6.767 | <0.0001 |
| Time from Baseline:A^-^T^-^N^+^ | 3.2 | 1.149 | 0.947 | 5.454 | 2.784 | 0.00538 |
| Time from Baseline:A^-^T^+^N^-^ | 4.077 | 1.487 | 1.163 | 6.992 | 2.743 | 0.00611 |
| Time from Baseline:A^+^T^-^N^-^ | 3.205 | 1.096 | 1.055 | 5.354 | 2.923 | 0.00348 |
| Time from Baseline:A^+^T^-^N^+^ | 12.994 | 1.975 | 9.121 | 16.866 | 6.578 | <0.0001 |
| Time from Baseline:A^+^T^+^N^-^ | 6.6 | 1.238 | 4.172 | 9.028 | 5.329 | <0.0001 |
| Time from Baseline:A^+^T^+^N^+^ | 9.003 | 0.76 | 7.513 | 10.492 | 11.849 | <0.0001 |
| Time from Baseline:Sex_Female_ | 0.391 | 0.409 | −0.411 | 1.194 | 0.956 | 0.33901 |
| A^-^T^-^N^+^:Sex_Female_ | 30.68 | 14.234 | 2.775 | 58.584 | 2.155 | 0.03118 |
| A^-^T^+^N^-^:Sex_Female_ | 14.26 | 19.521 | −24.009 | 52.529 | 0.731 | 0.46511 |
| A^+^T^-^N^-^:Sex_Female_ | −5.419 | 13.6 | −32.08 | 21.242 | −0.398 | 0.69029 |
| A^+^T^-^N^+^:Sex_Female_ | 26.261 | 19.189 | −11.358 | 63.88 | 1.369 | 0.17121 |
| A^+^T^+^N^-^:Sex_Female_ | 15.096 | 10.878 | −6.23 | 36.423 | 1.388 | 0.16528 |
| A^+^T^+^N^+^:Sex_Female_ | −8.311 | 10.31 | −28.523 | 11.902 | −0.806 | 0.42024 |
| Time from Baseline:A^-^T^-^N^+^:Sex_Female_ | −1.798 | 1.55 | −4.837 | 1.242 | −1.16 | 0.24625 |
| Time from Baseline:A^-^T^+^N^-^:Sex_Female_ | −0.764 | 1.792 | −4.277 | 2.748 | −0.427 | 0.66969 |
| Time from Baseline:A^+^T^-^N^-^:Sex_Female_ | −3.074 | 1.337 | −5.695 | −0.454 | −2.3 | 0.02149 |
| Time from Baseline:A^+^T^-^N^+^:Sex_Female_ | −1.627 | 2.59 | −6.705 | 3.451 | −0.628 | 0.52999 |
| Time from Baseline:A^+^T^+^N^-^:Sex_Female_ | −1.774 | 1.596 | −4.902 | 1.355 | −1.111 | 0.26648 |
| Time from Baseline:A^+^T^+^N^+^:Sex_Female_ | 11.036 | 1.61 | 7.879 | 14.193 | 6.853 | <0.0001 |

ATN: Categorical variable determining amyloid, tau, and neurodegeneration positivity status. Age_bl_ : Age at baseline. ID: Categorical variable indicating participant ID. Trail_B: Trail making test part B.

**Supplementary Table 12.** Model 2 estimates: **Animals ~** **Time from Baseline : ATN : Sex** + Time from Baseline : ATN + Time from Baseline + ATN + Time from Baseline : Sex + ATN : Sex + Sex + Age_bl_ + Education + (1|ID)

| Parameter | Coefficient | SE | CI_low | CI_high | T stat | P value |
| --- | --- | --- | --- | --- | --- | --- |
| (Intercept) | −20.01 | 1.645 | −23.235 | −16.786 | −12.165 | <0.0001 |
| Age_bl_ | 0.104 | 0.02 | 0.065 | 0.143 | 5.222 | <0.0001 |
| Time from Baseline | 0.149 | 0.032 | 0.087 | 0.211 | 4.702 | <0.0001 |
| A^-^T^-^N^+^ | 2.118 | 0.824 | 0.502 | 3.734 | 2.569 | 0.01023 |
| A^-^T^+^N^-^ | 0.393 | 1.514 | −2.574 | 3.36 | 0.26 | 0.7952 |
| A^+^T^-^N^-^ | 2.602 | 0.91 | 0.818 | 4.386 | 2.859 | 0.0042 |
| A^+^T^-^N^+^ | 2.958 | 1.125 | 0.753 | 5.162 | 2.63 | 0.0085 |
| A^+^T^+^N^-^ | 4.447 | 0.681 | 3.112 | 5.781 | 6.532 | <0.0001 |
| A^+^T^+^N^+^ | 7.876 | 0.583 | 6.734 | 9.019 | 13.518 | <0.0001 |
| Sex_Female_ | −0.763 | 0.496 | −1.736 | 0.21 | −1.538 | 0.12419 |
| Education | −0.504 | 0.057 | −0.615 | −0.394 | −8.927 | <0.0001 |
| Time from Baseline:A^-^T^-^N^+^ | 0.35 | 0.101 | 0.152 | 0.547 | 3.471 | 0.00052 |
| Time from Baseline:A^-^T^+^N^-^ | 0.325 | 0.144 | 0.043 | 0.608 | 2.257 | 0.02403 |
| Time from Baseline:A^+^T^-^N^-^ | 0.132 | 0.102 | −0.067 | 0.332 | 1.299 | 0.19407 |
| Time from Baseline:A^+^T^-^N^+^ | 0.598 | 0.181 | 0.243 | 0.953 | 3.306 | 0.00095 |
| Time from Baseline:A^+^T^+^N^-^ | 0.554 | 0.105 | 0.35 | 0.759 | 5.305 | <0.0001 |
| Time from Baseline:A^+^T^+^N^+^ | 0.51 | 0.07 | 0.372 | 0.648 | 7.243 | <0.0001 |
| Time from Baseline:Sex_Female_ | −0.068 | 0.039 | −0.145 | 0.009 | −1.731 | 0.08342 |
| A^-^T^-^N^+^:Sex_Female_ | 1.642 | 1.317 | −0.94 | 4.223 | 1.247 | 0.2125 |
| A^-^T^+^N^-^:Sex_Female_ | 1.405 | 1.871 | −2.262 | 5.073 | 0.751 | 0.45256 |
| A^+^T^-^N^-^:Sex_Female_ | −2.533 | 1.201 | −4.888 | −0.177 | −2.108 | 0.03507 |
| A^+^T^-^N^+^:Sex_Female_ | 2.831 | 1.739 | −0.578 | 6.24 | 1.628 | 0.10356 |
| A^+^T^+^N^-^:Sex_Female_ | 1.282 | 0.932 | −0.546 | 3.109 | 1.375 | 0.16926 |
| A^+^T^+^N^+^:Sex_Female_ | 0.411 | 0.835 | −1.225 | 2.048 | 0.493 | 0.62208 |
| Time from Baseline:A^-^T^-^N^+^:Sex_Female_ | −0.252 | 0.136 | −0.518 | 0.015 | −1.851 | 0.0642 |
| Time from Baseline:A^-^T^+^N^-^:Sex_Female_ | −0.263 | 0.171 | −0.598 | 0.072 | −1.538 | 0.12422 |
| Time from Baseline:A^+^T^-^N^-^:Sex_Female_ | −0.205 | 0.124 | −0.449 | 0.039 | −1.648 | 0.09943 |
| Time from Baseline:A^+^T^-^N^+^:Sex_Female_ | 0.176 | 0.231 | −0.276 | 0.629 | 0.764 | 0.44506 |
| Time from Baseline:A^+^T^+^N^-^:Sex_Female_ | −0.156 | 0.142 | −0.435 | 0.122 | −1.1 | 0.27156 |
| Time from Baseline:A^+^T^+^N^+^:Sex_Female_ | 0.722 | 0.132 | 0.463 | 0.98 | 5.475 | <0.0001 |

ATN: Categorical variable determining amyloid, tau, and neurodegeneration positivity status. Age_bl_ : Age at baseline. ID: Categorical variable indicating participant ID.

**Supplementary Table 13.** Model 2 estimates: **Vegetables ~** **Time from Baseline : ATN : Sex** + Time from Baseline : ATN + Time from Baseline + ATN + Time from Baseline : Sex + ATN : Sex + Sex + Age_bl_ + Education + (1|ID)

| Parameter | Coefficient | SE | CI_low | CI_high | T stat | P value |
| --- | --- | --- | --- | --- | --- | --- |
| (Intercept) | −13.69 | 1.19 | −16.024 | −11.357 | −11.501 | <0.0001 |
| Age_bl_ | 0.071 | 0.014 | 0.043 | 0.099 | 4.963 | <0.0001 |
| Time from Baseline | 0.104 | 0.025 | 0.055 | 0.154 | 4.113 | <0.0001 |
| A^-^T^-^N^+^ | 2.427 | 0.599 | 1.254 | 3.601 | 4.055 | <0.0001 |
| A^-^T^+^N^-^ | 0.567 | 1.1 | −1.589 | 2.723 | 0.516 | 0.606 |
| A^+^T^-^N^-^ | 1.894 | 0.661 | 0.598 | 3.189 | 2.866 | 0.00417 |
| A^+^T^-^N^+^ | 3.42 | 0.819 | 1.815 | 5.025 | 4.177 | <0.0001 |
| A^+^T^+^N^-^ | 3.602 | 0.495 | 2.632 | 4.572 | 7.281 | <0.0001 |
| A^+^T^+^N^+^ | 5.479 | 0.424 | 4.647 | 6.311 | 12.908 | <0.0001 |
| Sex_Female_ | −3.249 | 0.359 | −3.953 | −2.545 | −9.046 | <0.0001 |
| Education | −0.264 | 0.041 | −0.345 | −0.184 | −6.457 | <0.0001 |
| Time from Baseline:A^-^T^-^N^+^ | 0.12 | 0.08 | −0.038 | 0.278 | 1.488 | 0.13688 |
| Time from Baseline:A^-^T^+^N^-^ | 0.026 | 0.116 | −0.201 | 0.254 | 0.228 | 0.81953 |
| Time from Baseline:A^+^T^-^N^-^ | 0.166 | 0.081 | 0.006 | 0.326 | 2.04 | 0.04143 |
| Time from Baseline:A^+^T^-^N^+^ | 0.138 | 0.145 | −0.146 | 0.422 | 0.954 | 0.34005 |
| Time from Baseline:A^+^T^+^N^-^ | 0.395 | 0.084 | 0.231 | 0.559 | 4.723 | <0.0001 |
| Time from Baseline:A^+^T^+^N^+^ | 0.388 | 0.056 | 0.278 | 0.499 | 6.879 | <0.0001 |
| Time from Baseline:Sex_Female_ | 0.043 | 0.031 | −0.019 | 0.104 | 1.358 | 0.17437 |
| A^-^T^-^N^+^:Sex_Female_ | 0.503 | 0.956 | −1.371 | 2.377 | 0.527 | 0.59851 |
| A^-^T^+^N^-^:Sex_Female_ | 1.072 | 1.358 | −1.591 | 3.734 | 0.789 | 0.43011 |
| A^+^T^-^N^-^:Sex_Female_ | −1.347 | 0.874 | −3.061 | 0.367 | −1.54 | 0.1235 |
| A^+^T^-^N^+^:Sex_Female_ | 2.113 | 1.263 | −0.364 | 4.59 | 1.673 | 0.09446 |
| A^+^T^+^N^-^:Sex_Female_ | 1.405 | 0.68 | 0.072 | 2.739 | 2.066 | 0.03891 |
| A^+^T^+^N^+^:Sex_Female_ | 1.782 | 0.613 | 0.581 | 2.984 | 2.909 | 0.00364 |
| Time from Baseline:A^-^T^-^N^+^:Sex_Female_ | −0.139 | 0.109 | −0.352 | 0.075 | −1.275 | 0.20227 |
| Time from Baseline:A^-^T^+^N^-^:Sex_Female_ | 0.069 | 0.137 | −0.2 | 0.339 | 0.504 | 0.6142 |
| Time from Baseline:A^+^T^-^N^-^:Sex_Female_ | −0.233 | 0.1 | −0.428 | −0.038 | −2.338 | 0.0194 |
| Time from Baseline:A^+^T^-^N^+^:Sex_Female_ | 0.215 | 0.185 | −0.147 | 0.577 | 1.164 | 0.24433 |
| Time from Baseline:A^+^T^+^N^-^:Sex_Female_ | −0.071 | 0.114 | −0.295 | 0.152 | −0.624 | 0.53297 |
| Time from Baseline:A^+^T^+^N^+^:Sex_Female_ | 0.405 | 0.106 | 0.197 | 0.613 | 3.819 | 0.00014 |

ATN: Categorical variable determining amyloid, tau, and neurodegeneration positivity status. Age_bl_ : Age at baseline. ID: Categorical variable indicating participant ID.
